# Interpretable machine learning model to predict 90-day radiographically confirmed pneumonia after chemotherapy initiation in non-Hodgkin lymphoma: development and internal validation of a single-center cohort

**DOI:** 10.3389/fmed.2025.1674896

**Published:** 2025-09-22

**Authors:** Zhanna Zhang, Manqi Su, Panruo Jiang, Xiaoxia Wang, Lingling Kong, Xiangmin Tong, Gongqiang Wu

**Affiliations:** ^1^Department of Hematology, Dongyang Hospital Affiliated with Wenzhou Medical University, Jinhua, China; ^2^Department of Central Laboratory, Affiliated Hangzhou First People’s Hospital, School of Medicine, WestLake University, Hangzhou, China

**Keywords:** non-Hodgkin lymphoma, pneumonia, machine learning, risk prediction, Shapley additive explanations

## Abstract

**Background:**

Radiographically confirmed pneumonia within 90 days of chemotherapy initiation is a frequent and clinically important complication in patients with non-Hodgkin lymphoma, yet interpretable tools for early individualized risk estimation are limited.

**Objective:**

To develop and internally validate an interpretable machine-learning model that predicts the 90-day risk of radiographically confirmed pneumonia after chemotherapy initiation in non-Hodgkin lymphoma.

**Methods:**

We retrospectively analyzed 205 chemotherapy-treated NHL patients. A two-step feature selection (LASSO followed by random-forest–based recursive feature elimination) identified four predictors: high-grade malignancy, drinking (alcohol use), estimated glomerular filtration rate (eGFR), and smoking. Five algorithms were trained and compared under a stratified 70/30 split (training *n* = 145; internal hold-out test set *n* = 60) with leakage-safe preprocessing (within-fold kNN imputation, SMOTE, and scaling). The gradient boosting machine (GBM) performed best and was interpreted using SHAP. A web-based prototype was implemented for research use only.

**Results:**

On the internal hold-out test set (*n* = 60), the GBM achieved an AUC of 0.855 (95% CI 0.746–0.964), an F1 score of 0.679, and a Brier score of 0.155. SHAP identified reduced eGFR, smoking, drinking, and high-grade malignancy as influential contributors; case-level waterfall and force plots enhanced transparency. These estimates reflect internal validation only and were obtained without systematic microbiological confirmation or standardized radiologic rescoring. Accordingly, performance may be optimistic, and real-world use is not advised pending temporal and multicenter external validation (with potential recalibration) and prospective evaluation.

**Conclusion:**

The interpretable GBM model demonstrated promising discrimination and calibration on an internal hold-out test set; however, clinical deployment requires temporal and multicenter external validation (as well as prospective assessment with potential recalibration). The accompanying web calculator is a research-only prototype and is not intended for clinical decision-making until such validation is completed.

## 1 Introduction

Non-Hodgkin lymphoma (NHL) is a heterogeneous group of lymphoid malignancies for which global incidence continues to rise, reaching an estimated 545,000 new cases and 260,000 deaths in 2020 (1, 2). The widespread use of immunochemotherapy regimens, such as R-CHOP, along with the introduction of targeted therapies, has significantly improved survival. However, infectious complications—particularly pneumonia—remain among the most common and clinically significant adverse events during chemotherapy. In elderly NHL patients treated with R-CHOP, pulmonary complications have been reported in up to 40 percent of cases, with approximately 10 percent experiencing severe infections (3). A previous study involving 229 newly diagnosed NHL patients reported that 91 (39.7%) developed bacterial pneumonia, including 76 with isolated respiratory tract infection and 15 with concurrent bacteremia (4). Despite its frequency, pneumonia is often underrecognized in the early stages due to insidious and nonspecific symptoms, which can delay diagnosis and treatment, thereby adversely affecting clinical outcomes (5, 6). Although pneumonia is clinically burdensome—especially during the first 90 days after chemotherapy initiation, when patients are most vulnerable, validated tools for early risk prediction in this critical period remain limited. Few studies have developed individualized prediction models tailored to this early and high-risk phase of treatment.

Pneumonia in patients with NHL most commonly occurs within the first 90 days after chemotherapy initiation. This period is typically marked by bone marrow suppression, disruption of mucosal barriers, and compromised immune function (6–8). Early identification of high-risk individuals during this timeframe is essential to guide timely preventive interventions, such as empiric antimicrobial therapy, antifungal prophylaxis, or chemotherapy dose adjustments. However, existing risk stratification tools, including the MASCC score, were not specifically designed to predict pneumonia in patients with NHL, highlighting a critical gap in early risk assessment (9).

Several predictive tools exist in oncology for infection-related adverse outcomes, but they target different questions than organ-specific pneumonia risk. Febrile neutropenia (FN) risk stratification models—such as the Multinational Association for Supportive Care in Cancer (MASCC) Risk Index, Talcott classification, and the Clinical Index of Stable Febrile Neutropenia (CISNE)—were designed to identify low- versus high-risk FN episodes and guide outpatient management, not to estimate a patient’s individualized probability of developing pneumonia in the early chemotherapy period (10–13). In parallel, risk scores for invasive fungal disease (e.g., invasive mold disease) have been proposed primarily for hematologic malignancies or transplant settings, but these focus on a fungal subset rather than the broader spectrum of bacterial, viral, and fungal pneumonia encountered during NHL chemotherapy (14, 15). Consequently, current tools leave a gap for early, organ-specific prediction of pneumonia risk in NHL.

Pneumonia warrants prioritization for several clinically relevant reasons. First, the lung is a common infection site in patients with hematologic malignancies receiving chemotherapy; pneumonia contributes disproportionately to ICU admissions and mortality compared with many other infection sites (16, 17). Second, early detection is actionable: timely imaging, empiric antimicrobial therapy, selective antifungal prophylaxis, vaccination strategies, and—when appropriate—chemotherapy dose adjustments can be targeted to those at highest risk (18). Third, pneumonia’s insidious early presentation in immunocompromised hosts underscores the need for individualized, pre-emptive risk estimation to avoid delays in diagnosis and treatment.

To address this need, we developed and validated a machine-learning-based model to predict radiographically confirmed pneumonia occurring within 90 days after chemotherapy initiation in patients with NHL. Key predictors were identified using random forest–based recursive feature elimination (RF-RFE), and five machine learning algorithms were constructed and compared. Among these, the gradient boosting machine (GBM) model demonstrated superior predictive performance. To enhance interpretability and clinical applicability, SHAP (Shapley Additive Explanations) was applied to explain model predictions at the individual level, and the final model was implemented as an interactive web-based tool (19). This study provides a practical and individualized solution for early risk assessment of pneumonia in patients with NHL undergoing chemotherapy.

## 2 Methods

### 2.1 Study design and data source

#### 2.1.1 Study setting and cohort

This retrospective study was conducted at Dongyang Hospital Affiliated with Wenzhou Medical University included consecutive patients with pathologically confirmed non-Hodgkin lymphoma (NHL) who initiated systemic chemotherapy between October 2018 and October 2024. Eligibility required receipt of at least one chemotherapy cycle and follow-up for at least 90 days after initiation or until the earlier occurrence of pneumonia or death. We excluded patients with radiographically confirmed pneumonia prior to the initiation of chemotherapy and those with incomplete baseline or follow-up data.

#### 2.1.2 Primary outcome

The primary outcome was radiographically confirmed pneumonia occurring within 90 days of chemotherapy initiation. Case ascertainment required (i) a new or progressive pulmonary infiltrate on chest radiography or computed tomography and (ii) ≥ 1 clinical criterion: new or worsened cough or sputum; fever; auscultatory findings consistent with consolidation (e.g., crackles/wet rales, bronchial breath sounds, or egophony); or an abnormal peripheral leukocyte count > 10 × 10^9^/L or < 4 × 10^9^/L (with or without left shift). Episodes without a new infiltrate (e.g., tracheobronchitis/URTI, COPD/asthma exacerbation) and non-infectious mimics (drug- or radiation-induced pneumonitis, cardiogenic pulmonary edema, diffuse alveolar hemorrhage, pulmonary embolism/infarction, tumor progression/lymphangitic carcinomatosis) were excluded. Both community-onset and hospital-onset episodes meeting this definition were eligible. Because the 2019 ATS/IDSA community-acquired pneumonia guideline excludes immunocompromised hosts, we adopted its radiographic–clinical framework for case ascertainment only and did not apply CAP-specific care pathways.

#### 2.1.3 Data availability for phenotyping

Microbiological data (e.g., culture/PCR panels) were not systematically collected, and imaging studies were not centrally reviewed or scored using a standardized severity metric; case ascertainment relied on clinical radiology reports according to the operational definition.

### 2.2 Variable collection and preprocessing

A total of 35 clinical variables were collected, covering demographic characteristics, disease status, comorbidities, treatment-related factors, and baseline laboratory results. All predictors were pre-specified and dichotomized to binary indicators (0/1) before modeling; no multilevel factors remained (20). Missing values were imputed using k-nearest neighbors (kNN) with k = 5; imputation models were fit on the training data (or within cross-validation folds) and then applied to held-out data only to avoid information leakage (21). Because kNN imputation can yield fractional values for originally binary predictors, those fields were post-processed using a 0.5 threshold to restore 0/1 coding (22). Robustness to the imputation hyperparameter was examined in an alternative specification by varying k (3 and 7). Since this specification differs from the primary pipeline (settings and implementation), absolute performance values are reported in Supplementary Table S8 and are not intended for direct comparison with the primary test-set results; qualitative conclusions were unchanged.

### 2.3 Statistical analysis (baseline comparisons)

Baseline characteristics were summarized as *n* (%) for categorical variables. For comparisons between the pneumonia and no-pneumonia groups (Table 1), group differences were assessed using two-sided tests (*α* = 0.05): Pearson’s chi-square test with Yates’ continuity correction, or Fisher’s exact test when any expected cell count was < 5. Missing values were excluded from hypothesis testing. All baseline variables were dichotomous for presentation; binary 0/1 coding was used for model development (Section 2.2).

**Table 1:** Baseline characteristics of patients with non-Hodgkin lymphoma (NHL) with and without pneumonia within 90 days after chemotherapy initiation.

Variable	Overall (*n* = 205, %)	No pneumonia (*n* = 126, %)	Pneumonia (*n* = 79, %)	*P*
Gender				0.005
Female	89 (43.4)	65 (51.6)	24 (30.4)	
Male	116 (56.6)	61 (48.4)	55 (69.6)	
Age				0.189
> 65 years	104 (50.7)	69 (54.8)	35 (44.3)	
≤ 65 years	101 (49.3)	57 (45.2)	44 (55.7)	
Ann arbor stage				0.006
I-II	67 (33.5)	50 (41.3)	17 (21.5)	
III-IV	133 (66.5)	71 (58.7)	62 (78.5)	
NCCN-IPI				0.219
0–3	132 (70.2)	85 (73.9)	47 (64.4)	
4–5	56 (29.8)	30 (26.1)	26 (35.6)	
Post-chemotherapy marrow suppression				0.702
No	154 (75.1)	93 (73.8)	61 (77.2)	
Yes	51 (24.9)	33 (26.2)	18 (22.8)	
Hepatitis B status				0.246
No	167 (81.5)	99 (78.6)	68 (86.1)	
Yes	38 (18.5)	27 (21.4)	11 (13.9)	
Smoking				< 0.001
No	139 (67.8)	105 (83.3)	34 (43.0)	
Yes	66 (32.2)	21 (16.7)	45 (57.0)	
Drinking				< 0.001
No	137 (66.8)	104 (82.5)	33 (41.8)	
Yes	68 (33.2)	22 (17.5)	46 (58.2)	
Family history of malignant tumor				0.512
No	168 (82.0)	101 (80.2)	67 (84.8)	
Yes	37 (18.0)	25 (19.8)	12 (15.2)	
BMI				0.788
≥ 20 kg/m^2^	165 (82.1)	103 (83.1)	62 (80.5)	
< 20 kg/m^2^	36 (17.9)	21 (16.9)	15 (19.5)	
Hypertension				1.000
No	138 (69.0)	84 (68.9)	54 (69.2)	
Yes	62 (31.0)	38 (31.1)	24 (30.8)	
Diabetes				0.624
No	171 (85.5)	106 (86.9)	65 (83.3)	
Yes	29 (14.5)	16 (13.1)	13 (16.7)	
High-grade malignancy				< 0.001
No	83 (40.5)	68 (54.0)	15 (19.0)	
Yes	122 (59.5)	58 (46.0)	64 (81.0)	
Treatment regimen				0.290
Non-R-CHOP	119 (58.0)	69 (54.8)	50 (63.3)	
R-CHOP	86 (42.0)	57 (45.2)	29 (36.7)	
WBC				0.557
≥ 3.5 × 109/L	168 (82.4)	105 (84.0)	63 (79.7)	
< 3.5 × 109/L	36 (17.6)	20 (16.0)	16 (20.3)	
Hb				0.403
> 100 g/L	168 (82.0)	106 (84.1)	62 (78.5)	
≤ 100 g/L	37 (18.0)	20 (15.9)	17 (21.5)	
ANC				0.911
≥ 1.5 × 109/L	181 (88.3)	112 (88.9)	69 (87.3)	
< 1.5 × 109/L	24 (11.7)	14 (11.1)	10 (12.7)	
ALC				0.157
≥ 0.7 × 109/L	155 (75.6)	100 (79.4)	55 (69.6)	
< 0.7 × 109/L	50 (24.4)	26 (20.6)	24 (30.4)	
RDW				0.779
≤ 0.145	154 (75.1)	96 (76.2)	58 (73.4)	
> 0.145	51 (24.9)	30 (23.8)	21 (26.6)	
PLT				0.968
≥ 100 × 109/L	178 (86.8)	110 (87.3)	68 (86.1)	
< 100 × 109/L	27 (13.2)	16 (12.7)	11 (13.9)	
MPV				0.645
≤ 10.0 fL	78 (38.0)	50 (39.7)	28 (35.4)	
> 10.0 fL	127 (62.0)	76 (60.3)	51 (64.6)	
PCT				0.167
≤ 0.28	174 (84.9)	103 (81.7)	71 (89.9)	
> 0.28	31 (15.1)	23 (18.3)	8 (10.1)	
eGFR				< 0.001
≥ 80 mL/min/1.73 m^2^	152 (74.1)	114 (90.5)	38 (48.1)	
< 80 mL/min/1.73 m^2^	53 (25.9)	12 (9.5)	41 (51.9)	
Ca				0.889
≥ 2.1 mmol/L	140 (68.3)	87 (69.0)	53 (67.1)	
< 2.1 mmol/L	65 (31.7)	39 (31.0)	26 (32.9)	
Fe				0.578
≥ 9 μmol/L	123 (60.0)	78 (61.9)	45 (57.0)	
< 9 μmol/L	82 (40.0)	48 (38.1)	34 (43.0)	
ALB				1.000
> 30 g/L	177 (86.3)	109 (86.5)	68 (86.1)	
≤ 30 g/L	28 (13.7)	17 (13.5)	11 (13.9)	
A/G ratio				0.650
≥ 1	183 (89.3)	111 (88.1)	72 (91.1)	
<1	22 (10.7)	15 (11.9)	7 (8.9)	
ALT				1.000
≤ 40 U/L	176 (85.9)	108 (85.7)	68 (86.1)	
> 40 U/L	29 (14.1)	18 (14.3)	11 (13.9)	
AST				0.577
≤ 40 U/L	181 (88.3)	113 (89.7)	68 (86.1)	
> 40 U/L	24 (11.7)	13 (10.3)	11 (13.9)	
LDH				0.329
≤ 222 U/L	104 (51.7)	67 (54.9)	37 (46.8)	
> 222 U/L	97 (48.3)	55 (45.1)	42 (53.2)	
α-HBDH				0.545
≤ 182 U/L	112 (56.6)	71 (58.7)	41 (53.2)	
> 182 U/L	86 (43.4)	50 (41.3)	36 (46.8)	
TG				1.000
≤ 1.7 mmol/L	157 (77.0)	96 (76.8)	61 (77.2)	
> 1.7 mmol/L	47 (23.0)	29 (23.2)	18 (22.8)	
HDL				0.799
≥ 1.0 mmol/L	81 (39.7)	51 (40.8)	30 (38.0)	
<1.0 mmol/L	123 (60.3)	74 (59.2)	49 (62.0)	
LDL				0.349
< 3.4 mmol/L	182 (89.2)	109 (87.2)	73 (92.4)	
≥ 3.4 mmol/L	22 (10.8)	16 (12.8)	6 (7.6)	
CRP				0.320
≤ 10 mg/L	115 (58.1)	73 (61.3)	42 (53.2)	
> 10 mg/L	83 (41.9)	46 (38.7)	37 (46.8)	

Values are *n* (%). *p* values were calculated using Pearson’s chi-square test with Yates’ continuity correction (or Fisher’s exact test when any expected cell count was < 5); two-sided *α* = 0.05. Missing values were excluded from hypothesis testing. Variables are dichotomized for presentation. Per-variable missingness is summarized in Supplementary Table S1.

For comparisons between the training and test sets (Table 2), standardized mean differences (SMDs) were reported as effect sizes that are less sensitive to sample size (imbalance flagged at |SMD| > 0.20; |SMD| < 0.10 generally negligible) (23–25). Train–test comparability was summarized with SMDs only, without *p* values. A pre-specified sensitivity analysis excluding variables with |SMD| > 0.20 yielded essentially unchanged results (Supplementary Tables S9, S10).

**Table 2:** Baseline characteristics of the training set and the internal hold-out test set (derived by stratified random split from the same single-center cohort).

Variable	Internal hold-out test set (*n* = 60, %)	Training set (*n* = 145, %)	SMD
Gender			0.045
Female	27 (45.00)	62 (42.76)	
Male	33 (55.00)	83 (57.24)	
Age			0.026
> 65 years	31 (51.67)	73 (50.34)	
≤ 65 years	29 (48.33)	72 (49.66)	
Ann arbor stage			0.176
I-II	17 (28.33)	53 (36.55)	
III-IV	43 (71.67)	92 (63.45)	
NCCN-IPI			0.125
0–3	40 (66.67)	105 (72.41)	
4–5	20 (33.33)	40 (27.59)	
Post-chemotherapy marrow suppression			0.058
No	44 (73.33)	110 (75.86)	
Yes	16 (26.67)	35 (24.14)	
Hepatitis B status			0.069
No	50 (83.33)	117 (80.69)	
Yes	10 (16.67)	28 (19.31)	
Smoking			0.067
No	42 (70.00)	97 (66.90)	
Yes	18 (30.00)	48 (33.10)	
Drinking			0.104
No	38 (63.33)	99 (68.28)	
Yes	22 (36.67)	46 (31.72)	
Family history of malignant tumor			0.071
No	48 (80.00)	120 (82.76)	
Yes	12 (20.00)	25 (17.24)	
BMI			0.149
≥ 20 kg/m^2^	47 (78.33)	122 (84.14)	
< 20 kg/m^2^	13 (21.67)	23 (15.86)	
Hypertension			0.079
No	40 (66.67)	102 (70.34)	
Yes	20 (33.33)	43 (29.66)	
Diabetes			0.100
No	50 (83.33)	126 (86.90)	
Yes	10 (16.67)	19 (13.10)	
High-grade malignancy			0.160
No	21 (35.00)	62 (42.76)	
Yes	39 (65.00)	83 (57.24)	
Treatment regimen			0.040
Non-R-CHOP	34 (56.67)	85 (58.62)	
R-CHOP	26 (43.33)	60 (41.38)	
WBC			0.208
≥ 3.5 × 109/L	46 (76.67)	123 (84.83)	
< 3.5 × 109/L	14 (23.33)	22 (15.17)	
Hb			0.071
> 100 g/L	48 (80.00)	120 (82.76)	
≤ 100 g/L	12 (20.00)	25 (17.24)	
ANC			0.209
≥ 1.5 × 109/L	50 (83.33)	131 (90.34)	
< 1.5 × 109/L	10 (16.67)	14 (9.66)	
ALC			0.181
≥ 0.7 × 109/L	42 (70.00)	113 (77.93)	
< 0.7 × 109/L	18 (30.00)	32 (22.07)	
RDW			0.004
≤ 0.145	45 (75.00)	109 (75.17)	
> 0.145	15 (25.00)	36 (24.83)	
PLT			0.207
≥ 100 × 109/L	49 (81.67)	129 (88.97)	
< 100 × 109/L	11 (18.33)	16 (11.03)	
MPV			0.008
≤ 10.0 fL	23 (38.33)	55 (37.93)	
> 10.0 fL	37 (61.67)	90 (62.07)	
PCT			0.072
≤ 0.28	52 (86.67)	122 (84.14)	
> 0.28	8 (13.33)	23 (15.86)	
eGFR			0.028
≥ 80 mL/min/1.73 m^2^	45 (75.00)	107 (73.79)	
< 80 mL/min/1.73 m^2^	15 (25.00)	38 (26.21)	
Ca			0.052
≥ 2.1 mmol/L	42 (70.00)	98 (67.59)	
< 2.1 mmol/L	18 (30.00)	47 (32.41)	
Fe			< 0.001
≥ 9 μmol/L	36 (60.00)	87 (60.00)	
< 9 μmol/L	24 (40.00)	58 (40.00)	
ALB			0.186
> 30 g/L	49 (81.67)	128 (88.28)	
≤ 30 g/L	11 (18.33)	17 (11.72)	
A/G ratio			0.034
≥ 1	54 (90.00)	129 (88.97)	
<1	6 (10.00)	16 (11.03)	
ALT			0.100
≤ 40 U/L	50 (83.33)	126 (86.90)	
> 40 U/L	10 (16.67)	19 (13.10)	
AST			0.140
≤ 40 U/L	51 (85.00)	130 (89.66)	
> 40 U/L	9 (15.00)	15 (10.34)	
LDH			0.013
≤ 222 U/L	31 (51.67)	74 (51.03)	
> 222 U/L	29 (48.33)	71 (48.97)	
α-HBDH			0.160
≤ 182 U/L	30 (50.00)	84 (57.93)	
> 182 U/L	30 (50.00)	61 (42.07)	
TG			0.043
≤ 1.7 mmol/L	47 (78.33)	111 (76.55)	
> 1.7 mmol/L	13 (21.67)	34 (23.45)	
HDL			0.014
≥ 1.0 mmol/L	24 (40.00)	57 (39.31)	
< 1.0 mmol/L	36 (60.00)	88 (60.69)	
LDL			0.116
< 3.4 mmol/L	52 (86.67)	131 (90.34)	
≥ 3.4 mmol/L	8 (13.33)	14 (9.66)	
CRP			0.392
≤ 10 mg/L	27 (45.00)	93 (64.14)	
> 10 mg/L	33 (55.00)	52 (35.86)	

Values are *n* (%). Standardized mean differences (SMDs) are reported as effect sizes; imbalance was flagged at |SMD| > 0.20 (|SMD| < 0.10 generally negligible). No *P* values are reported, consistent with recommendations to reduce sample-size dependence.

Analyses were performed in R (version 4.4.2) using the TableOne package (CreateTableOne) and base R functions. Methods for AUC (DeLong), bootstrap 95% CIs for threshold-based metrics, calibration, and decision-curve analysis are detailed in Section 2.7.

### 2.4 Data split and internal validation

We performed a stratified 70/30 split based on the outcome (pneumonia vs. no pneumonia) to create a training set (*n* = 145) and an internal hold-out test set (*n* = 60) from the same single-center cohort, ensuring balanced class distributions across sets. All preprocessing steps—including kNN imputation, feature selection (LASSO followed by RF-RFE), class-imbalance handling (SMOTE within cross-validation), and hyperparameter tuning—were conducted strictly on the training data. These procedures were confined to cross-validation folds to prevent information leakage. The hold-out test set was left untouched until final evaluation. Because the internal hold-out test set was modest (*n* = 60; events = 23), we treated performance from repeated 10-fold cross-validation in the training set as the primary estimate, while test-set metrics served as complementary internal validation and are reported with 95% CIs (AUC via DeLong; other metrics via class-stratified bootstrap, B = 2,000).

**Figure 1:**
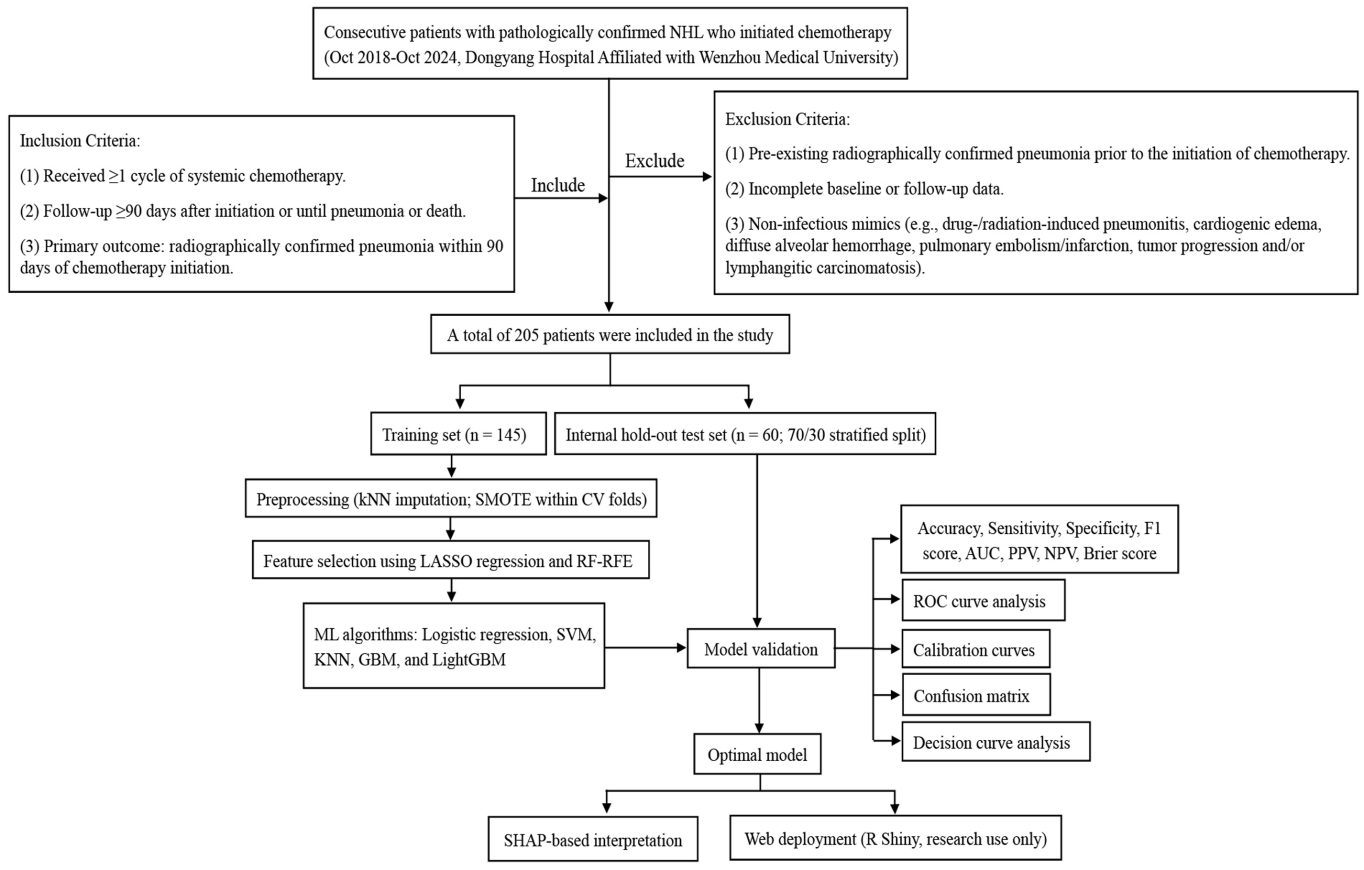
Flowchart of patient selection and machine-learning workflow for predicting radiographically confirmed pneumonia within 90 days after chemotherapy initiation in NHL patients.

Baseline comparability between the training and test sets was summarized using standardized mean differences (SMDs) and is presented in Table 2. We did not report hypothesis-test *p* values for the train-test comparison because such tests are highly sample-size dependent and less informative for balance assessment in a small internal test set. Importantly, the internal hold-out test set was never resampled and was kept completely isolated from all preprocessing, imputation, SMOTE, and feature-selection procedures (see Figure 1).

### 2.5 Feature selection

**Figure 2:**
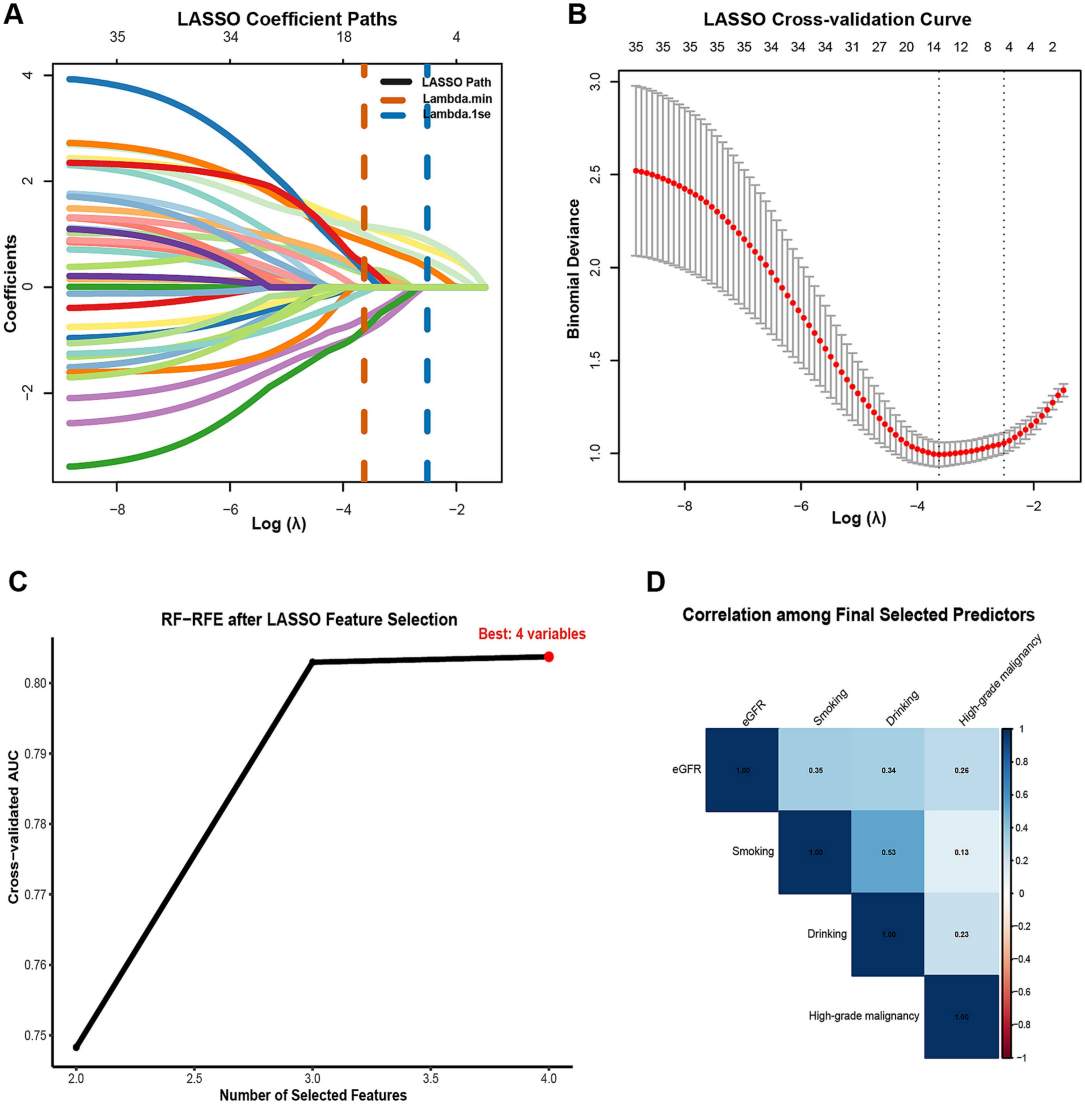
Two-stage feature selection for predicting radiographically confirmed pneumonia within 90 days after chemotherapy initiation in NHL patients. **(A)** LASSO coefficient profiles across Log Lambda; vertical dashed lines mark Lambda min (orange) and Lambda 1se (blue). Numbers along the top axis indicate the count of non-zero coefficients at each Lambda. **(B)** LASSO 10-fold cross-validation curve showing mean binomial deviance (red) with SE bars versus Log Lambda; the parsimonious Lambda 1se was adopted for variable retention. **(C)** Cross-validated AUC by subset size during EPV-constrained RF-RFE after LASSO selection; the optimal subset comprised four predictors. **(D)** Correlation among the final selected predictors (eGFR, smoking, drinking, high-grade malignancy). numeric collinearity diagnostics (VIF/GVIF) are provided in Supplementary Table S5. Feature selection and tuning were performed strictly within training cross-validation folds; the internal hold-out test set was not used for selection.

To identify a parsimonious and stable set of predictors, we used a two-step procedure. First, least absolute shrinkage and selection operator (LASSO) logistic regression was applied to the full set of 35 candidate variables with 10-fold cross-validation; to favor sparsity and reduce variance, we selected the *λ*.1se solution (26). In our data, LASSO (λ.1se) retained four variables—high-grade malignancy, drinking, eGFR, and smoking. Second, we performed events-per-variable (EPV)—constrained random-forest–based recursive feature elimination (RF-RFE) on the LASSO-retained variables, enforcing EPV ≥ 5 and a maximum of 10 predictors, which was appropriate for the 79 pneumonia events (27). We prespecified a stopping rule to select the smallest subset on the performance plateau, defined as ∆AUC ≤ 0.01 from the maximal cross-validated AUC. When adjacent subsets both satisfied the plateau criterion, a pre-specified tie-breaker favored the subset with the higher mean CV AUC and greater selection stability; this yielded the final four-predictor model. The incremental AUC-by-subset curve is shown in (Figure 2C), and stepwise subsets are tabulated in (Supplementary Table S2). To assess robustness, we conducted nested cross-validation (outer 5-fold, 20 repeats): within each outer training fold, LASSO (*λ*.1se) and EPV-constrained RFE were refit, and performance was evaluated on the corresponding outer test fold; selection frequencies across repeats were summarized (Supplementary Figure 1A; Supplementary Table S3). In addition, we performed 200 bootstrap resamples of the LASSO step (λ.1se) to estimate per-variable selection probabilities (Supplementary Table S4). Potential multicollinearity among final predictors was screened using Pearson correlations (after dummy expansion) and variance inflation factors (VIF/GVIF); we flagged |r| > 0.70 or VIF > 5 (GVIF_adj > 2) as concerning. The correlation heatmap appears in (Figure 2D), with numeric diagnostics in (Supplementary Table S5).

### 2.6 Hyperparameter tuning and model selection

Hyperparameter tuning was performed exclusively on the training set to avoid information leakage. For the support vector machine (SVM), GBM, and k-nearest neighbors (KNN), we employed Bayesian optimization (rBayesianOptimization, upper confidence bound acquisition with *κ* = 2.0). Each candidate hyperparameter configuration was evaluated using 5-fold cross-validation, with SMOTE applied strictly within folds. The binary outcome was coded as pneumonia = “Yes” and no pneumonia = “No”; model selection optimized the mean cross-validated AUC (caret twoClassSummary, positive = “Yes”). For SVM and KNN only, predictors were z-score centered and scaled during both the 5-fold tuning stage and the subsequent repeated 10-fold cross-validation stage used for performance estimation and final refitting; scaling parameters were estimated on the in-fold training (analysis) partition and applied to the paired validation (assessment) partition, with no carryover across folds. After model selection, the centering/scaling transform was refit on the full training set and applied once to the internal hold-out test set before final evaluation. Other models (logistic regression, GBM, LightGBM) did not use feature scaling. Logistic regression did not require hyperparameter tuning.

For the light gradient boosting machine (LightGBM), a two-stage procedure was adopted. First, Bayesian search with 4-fold cross-validation and early stopping was performed across predefined ranges for learning rate, num_leaves, min_data_in_leaf, feature_fraction, bagging_fraction, λ1, and λ2. Second, the optimal parameter set underwent confirmatory 5-fold cross-validation to determine the best iteration number, after which the final model was refitted on the full training set for nrounds = best_iter. The internal hold-out test set was never used for resampling, tuning, or threshold determination. Detailed search bounds and final selected hyperparameter values for all models are provided in Supplementary Table S6.

### 2.7 Model development and comparison

To mitigate class imbalance during model development, we applied SMOTE within each resampling fold of cross-validation using caret:trainControl (sampling = “smote”). The same within-fold procedure was used during hyperparameter tuning. No resampling was ever applied to the internal hold-out test set. By design, this prevents information leakage and yields unbiased validation estimates (28). Five machine-learning models were constructed based on the selected predictors: logistic regression, SVM, KNN, GBM, and LightGBM. Hyperparameters were optimized via Bayesian optimization evaluated with 5-fold cross-validation on the training set (for LightGBM: 4-fold during the search with early stopping plus confirmatory 5-fold to determine best_iter), as detailed in Section 2.6. Repeated 10-fold cross-validation (10 × 5) on the training set was then used for performance estimation and to generate out-of-fold predictions; the internal hold-out test set was used once for final evaluation. Model performance was evaluated on the internal hold-out test set using multiple metrics, including the AUC, F1 score, Brier score, accuracy, sensitivity, specificity, and predictive values. Decision thresholds were pre-specified on the training set by maximizing Youden’s J and then fixed for both training and test evaluations. We report AUC with DeLong 95% confidence intervals (CIs); at the fixed threshold we report accuracy, sensitivity, specificity, PPV, NPV, and F1 score with class-stratified bootstrap 95% CIs (B = 2,000), together with confusion-matrix counts (TP, TN, FP, FN). Calibration was assessed using calibration curves and the Brier score with bootstrap 95% CIs, and clinical utility was evaluated using decision-curve analysis (DCA) on predicted probabilities. All preprocessing resampling (SMOTE), hyperparameter tuning, and threshold selection were confined to training-set cross-validation; the test set was used only once for final evaluation.

### 2.8 Model interpretation

To enhance the interpretability of the final GBM model, a *post hoc* analysis was conducted using SHAP, a game-theoretic approach that quantifies the contribution of individual predictors to the model outputs. Global feature importance was visualized using a SHAP summary bar plot and a beeswarm plot, which illustrate the relative magnitude and direction of each variable’s impact on the predicted risk of pneumonia. For individual-level interpretation, a SHAP waterfall plot and a force plot were generated to demonstrate how specific predictor values influenced the deviation from the model’s baseline prediction, thereby providing transparent, case-specific insights into the model’s decision-making process (29, 30).

### 2.9 Web deployment

The final GBM model was implemented as an interactive R Shiny web application for research use. This platform accepts patient-specific clinical data and computes risk estimates with explanations for research evaluation. The tool is a prototype for internal validation and has not undergone prospective clinical implementation or impact evaluation; it is not intended to guide patient care or clinical decision-making and has not been integrated into routine clinical workflows (31, 32).

## 3 Results

### 3.1 Baseline characteristics

A total of 205 patients diagnosed with NHL were included in the final analysis, among whom 79 (38.5%) developed pneumonia within 90 days after chemotherapy initiation. Baseline characteristics were compared between the pneumonia group (*n* = 79) and the no-pneumonia group (*n* = 126) (Table 1). Patients with pneumonia were more often male (69.6% vs. 48.4%, *p* = 0.005) and more frequently had advanced Ann Arbor stage III–IV disease (78.5% vs. 58.7%, *p* = 0.006). Significant differences were also observed in smoking (57.0% vs. 16.7%, *p* < 0.001), drinking (58.2% vs. 17.5%, *p* < 0.001), high-grade malignancy (81.0% vs. 46.0%, *p* < 0.001), and reduced renal function (eGFR < 80 mL/min/1.73 m^2^, 51.9% vs. 9.5%, *p* < 0.001). In contrast, there were no statistically significant differences between groups for age (*p* = 0.189), NCCN-IPI (*p* = 0.219), post-chemotherapy marrow suppression (*p* = 0.702), hepatitis B status (*p* = 0.246), BMI (*p* = 0.788), hypertension (*p* = 1.000), diabetes (*p* = 0.624), treatment regimen (*p* = 0.290), or the majority of baseline hematologic/biochemical indices (all *p* > 0.05), including WBC, Hb, ANC, ALC, RDW, PLT, MPV, PCT, calcium, iron, albumin, A/G ratio, ALT, AST, LDH, *α*-HBDH, triglycerides, HDL-C, LDL-C, and CRP.

For model development, we then performed a stratified 70/30 split by the outcome to create a training set (*n* = 145) and an internal hold-out test set (*n* = 60) from the same single-center cohort. Between-set comparability was summarized using standardized mean differences (SMDs) (Table 2). Most characteristics were well balanced (|SMD| < 0.20). Four laboratory variables—WBC, ANC, PLT, and CRP—showed modest imbalance (|SMD| ≈ 0.21, 0.21, 0.21, and 0.39, respectively). A prespecified sensitivity analysis excluding these variables is described in Section 3.4 and Supplementary Tables S9, S10.

### 3.2 Feature selection results

Using the pre-specified two-step procedure, LASSO (*λ*.1se) retained four variables—high-grade malignancy, drinking, eGFR, and smoking (Figures 2A,B). Applying events-per-variable–constrained RF-RFE to this set, the cross-validated AUC increased with the number of features and reached a performance plateau at 3–4 predictors (Figure 2C). In our data, the mean cross-validation (CV) AUCs were 0.803 for k = 3 and 0.804 for k = 4 (∆AUC = 0.001, within the pre-specified plateau tolerance ∆AUC ≤ 0.01; Supplementary Table S2). Per our tie-breaker for adjacent subsets on the plateau—which selected the subset with the higher mean CV AUC and greater selection stability—we retained the four-predictor model (high-grade malignancy, drinking, eGFR, smoking) for subsequent development and interpretation. Stability was high in nested cross-validation (outer 5-fold, 20 repeats), with consistently elevated selection frequencies for these four predictors (Supplementary Table S3; Supplementary Figure S1A). Bootstrap resampling of the LASSO step (B = 200) produced concordant stability results (Supplementary Table S4). Collinearity diagnostics (VIF/GVIF and Pearson correlations) indicated no evidence of concerning multicollinearity among the final predictors (Figure 2D; Supplementary Table S5).

### 3.3 Model performance comparison

Model performance was reported at a pre-specified threshold fixed from the training set by Youden’s J. All threshold-based metrics were presented with class-stratified bootstrap 95% CIs; AUC 95% CIs were DeLong-based. Five machine-learning models—logistic regression, SVM, KNN, GBM, and LightGBM—were all trained using the same four selected predictors, avoiding model-specific reselection and potential selection-induced bias. The performance of each model was subsequently evaluated on both the training set and an internal hold-out test set (*n* = 60).

**Figure 3:**
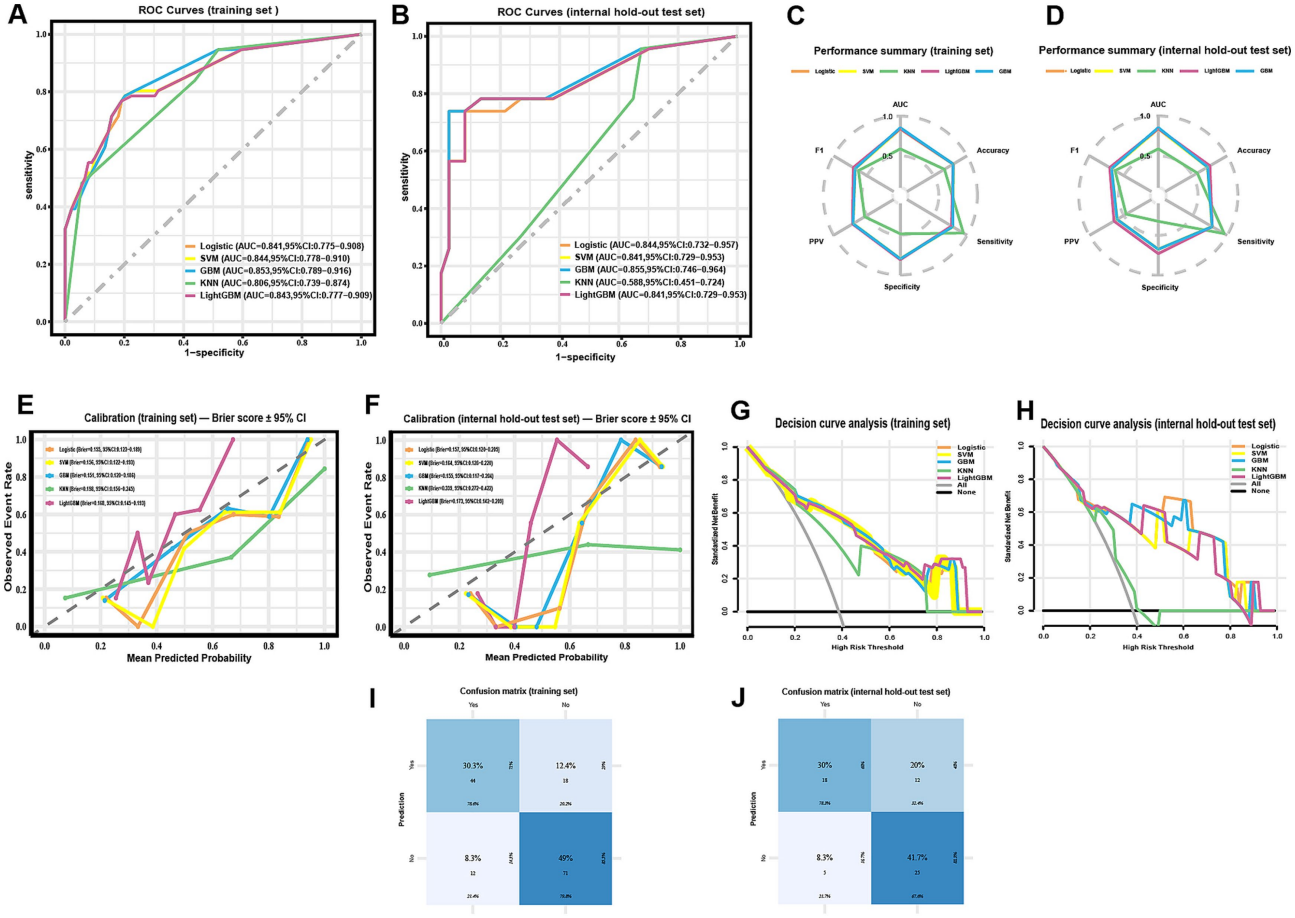
Comparative performance of five machine-learning models for predicting radiographically confirmed pneumonia within 90 days after chemotherapy initiation in NHL patients. **(A,B)** ROC curves for the training set **(A)** and the internal hold-out test set **(B)**; AUCs with DeLong 95% CIs are reported in the legends. **(C,D)** Radar plots summarizing threshold-based metrics (AUC, accuracy, sensitivity, specificity, PPV, F1) for the training set **(C)** and the internal hold-out test set **(D)** at the pre-specified threshold (Youden’s J, determined from the training set and fixed for testing); corresponding 95% CIs are provided in Table 3. **(E,F)** Calibration curves with corresponding Brier scores (bootstrap 95% CIs) for the training set **(E)** and the internal hold-out test set **(F)**. **(G,H)** Decision-curve analysis in the training set **(G)** and the internal hold-out test set **(H)**, showing net benefit across threshold probabilities; “all” and “none” strategies are included for reference. **(I,J)** Confusion matrices of the GBM model for the training set **(I)** and the internal hold-out test set (**J**; *n* = 60) at the pre-specified threshold. ROC CIs are DeLong-based; threshold-based metrics and Brier CIs use class-stratified bootstrap. All preprocessing, feature selection, SMOTE, and tuning were confined to training cross-validation; the internal test set was not used for resampling or threshold selection.

On the training set, GBM achieved the highest AUC (0.853, 95% CI 0.789–0.916), followed by SVM (0.844, 95% CI 0.778–0.910), LightGBM (0.843, 95% CI 0.777–0.909), logistic regression (0.841, 95% CI 0.775–0.908), and KNN (0.806, 95% CI 0.739–0.874) (Figure 3A). On the internal hold-out test set, GBM achieved the highest AUC (0.855, 95% CI 0.746–0.964), followed by logistic regression (0.844, 95% CI 0.732–0.957), LightGBM and SVM (both 0.841, 95% CI 0.729–0.953), and KNN (0.588, 95% CI 0.451–0.724) (Figure 3B). These results indicate that GBM provided the best overall discrimination in both training cross-validation and internal testing.

Full threshold-based metrics with 95% CIs and the corresponding confusion-matrix counts (TP/TN/FP/FN) for all models are provided in Table 3. For completeness, training-set metrics with 95% CIs are provided in Supplementary Table S7. As illustrated in the radar plots (Figures 3C–D), GBM outperformed the other algorithms in terms of overall discrimination (AUC) and achieved balanced sensitivity and predictive values. LightGBM yielded slightly higher accuracy (0.750 vs. 0.717) and specificity (0.730 vs. 0.676), but GBM maintained the most robust overall performance across metrics. Calibration curves demonstrated good agreement between predicted and observed risks for GBM, with Brier scores of 0.151 (training) and 0.155 (internal test) (Figures 3E–F; Table 3). Decision-curve analysis showed comparable net benefit for GBM, logistic regression, SVM, and LightGBM across most clinically relevant thresholds, with no uniform winner; KNN underperformed (Figures 3G–H). These patterns support decision-analytic utility under internal validation. The confusion matrices further illustrate the classification performance of the GBM model in both the training set (Figure 3I) and the internal hold-out test set (Figure 3J). In the training set, correct predictions predominated, with relatively balanced error rates across positive and negative classes. In the test set, although overall discrimination was maintained, a higher proportion of false positives was observed, reflecting a modest decrease in specificity.

**Table 3:** Performance of machine-learning models on the internal hold-out test set using pre-specified training-set thresholds.

Model	Threshold	Accuracy (95% CI)	Sensitivity (95% CI)	Specificity (95% CI)	PPV (95% CI)	NPV (95% CI)	F1 (95% CI)	AUC (95% CI)	TP	TN	FP	FN
Logistic	0.386	0.717 (0.600–0.817)	0.783 (0.609–0.957)	0.676 (0.514–0.811)	0.600 (0.484–0.731)	0.833 (0.714–0.957)	0.679 (0.545–0.792)	0.844 (0.732–0.957)	18	25	12	5
SVM	0.398	0.717 (0.600–0.817)	0.783 (0.609–0.957)	0.676 (0.514–0.811)	0.600 (0.484–0.731)	0.833 (0.714–0.957)	0.679 (0.545–0.792)	0.841 (0.729–0.953)	18	25	12	5
GBM	0.418	0.717 (0.600–0.817)	0.783 (0.609–0.957)	0.676 (0.514–0.811)	0.600 (0.484–0.731)	0.833 (0.714–0.957)	0.679 (0.545–0.792)	0.855 (0.746–0.964)	18	25	12	5
KNN	0.167	0.567 (0.467–0.667)	0.957 (0.870–1.000)	0.324 (0.189–0.486)	0.468 (0.413–0.537)	0.923 (0.750–1.000)	0.629 (0.563–0.697)	0.588 (0.451–0.724)	22	12	25	1
LightGBM	0.386	0.750 (0.633–0.850)	0.783 (0.609–0.957)	0.730 (0.568–0.865)	0.643 (0.516–0.783)	0.844 (0.735–0.962)	0.706 (0.571–0.824)	0.841 (0.729–0.953)	18	27	10	5

Thresholds were pre-specified on the training set by maximizing Youden’s J (positive class = “yes”) and fixed for evaluation on the internal hold-out test set. AUC 95% confidence intervals (CIs) were estimated using the DeLong method; other 95% CIs for threshold-based metrics were obtained by class-stratified bootstrap (B = 2,000). Counts are TP/TN/FP/FN at the fixed threshold.

Collectively, these findings support GBM as the most robust model for individualized pneumonia-risk prediction under internal validation. All estimates reflect internal validation only; in the absence of routine microbiological confirmation, standardized radiologic scoring, and any real-world/prospective validation, performance may be optimistic and not ready for clinical deployment.

### 3.4 Sensitivity analysis of imputation

To assess robustness to imputation, we examined an alternative specification that varied the k parameter in kNN imputation (k = 3, 5, and 7). Because this specification differed from the primary pipeline, absolute performance values were presented in Supplementary Table S8 and should not be interpreted as head-to-head comparisons with the primary test-set results. Results were qualitatively unchanged. Second, to address modest baseline imbalances, we repeated the primary pipeline without changes (i.e., same preprocessing, cross-validation/tuning protocol, and model class) but excluded variables with |SMD| > 0.20 between the training and test sets (WBC, ANC, PLT, CRP). Performance was essentially unchanged relative to the primary analysis (Supplementary Tables S9, S10), indicating that these variables did not materially influence model performance.

### 3.5 SHAP-based model interpretation

**Figure 4:**
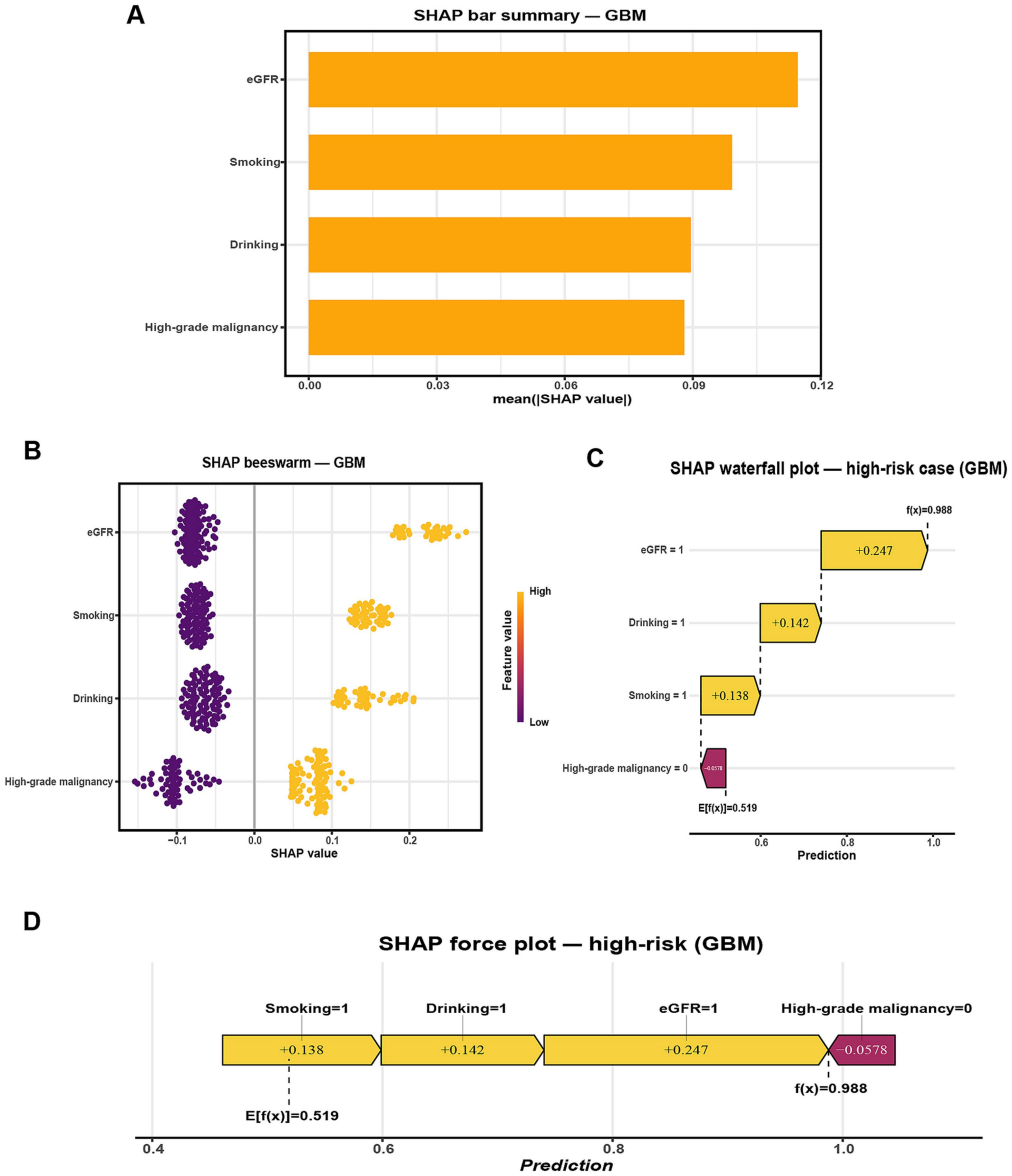
SHAP-based interpretation of the GBM model for predicting radiographically confirmed pneumonia within 90 days after chemotherapy initiation in NHL patients. **(A)** Global feature importance ranked by mean absolute SHAP value. **(B)** SHAP beeswarm plot showing the distribution, direction, and magnitude of feature contributions across the internal hold-out test set (points colored by feature value). **(C)** SHAP waterfall plot for a representative high-risk case (Smoking = 1, Drinking = 1, eGFR = 1, High-grade malignancy = 0), illustrating how individual feature contributions shift the predicted probability from the baseline E[f(x)] = 0.519 to f(x) = 0.988. **(D)** SHAP force plot for the same case, summarizing how these contributions combine to yield the final prediction. Predictor encoding: Smoking (1 = Yes, 0 = No), Drinking (1 = Yes, 0 = No), High-grade malignancy (1 = Yes, 0 = No), eGFR (1 = < 80 mL/min/1.73 m^2^, 0 = ≥ 80 mL/min/1.73 m^2^).

To improve the interpretability of the GBM model, SHAP was applied to quantify the contribution of each predictor to the model’s output. The SHAP summary bar plot (Figure 4A) ranked the four selected features by their mean absolute SHAP values, with eGFR showing the strongest overall influence, followed by smoking, drinking, and high-grade malignancy. The SHAP beeswarm plot (Figure 4B) visualized the distribution and direction of each feature’s contribution. Generally, higher SHAP values for high-grade malignancy and lower eGFR were associated with increased predicted risk of pneumonia, while the effects of smoking and drinking varied across individuals.

For case-level interpretation, waterfall and force plots (Figures 4C,D) were generated for a representative high-risk patient. In this example, concurrent smoking, drinking, and reduced renal function substantially elevated the model’s predicted risk despite the absence of high-grade malignancy, as reflected by a high model output (f(x) = 0.988 vs. E[f(x)] = 0.519). To complement this, an illustrative low-risk case was presented in Supplementary Figures S1B,C, where the absence of high-grade malignancy, preserved renal function (eGFR = 0), and lack of smoking and drinking collectively reduced the predicted probability well below the baseline expectation (f(x) = 0.186 vs. E[f(x)] = 0.519).

These individualized explanations improve transparency, highlight clinically actionable factors, and provide practical insights for prevention. For example, smoking cessation and alcohol avoidance represent modifiable targets, while vigilant monitoring of renal function and high-grade disease may guide early intervention. Together, these case-level insights support patient-centered decision-making and strengthen the clinical applicability of the model. Such individualized visualizations enhance model transparency and reinforce personalized clinical decision-making.

### 3.6 Web-based prediction tool deployment

**Figure 5:**
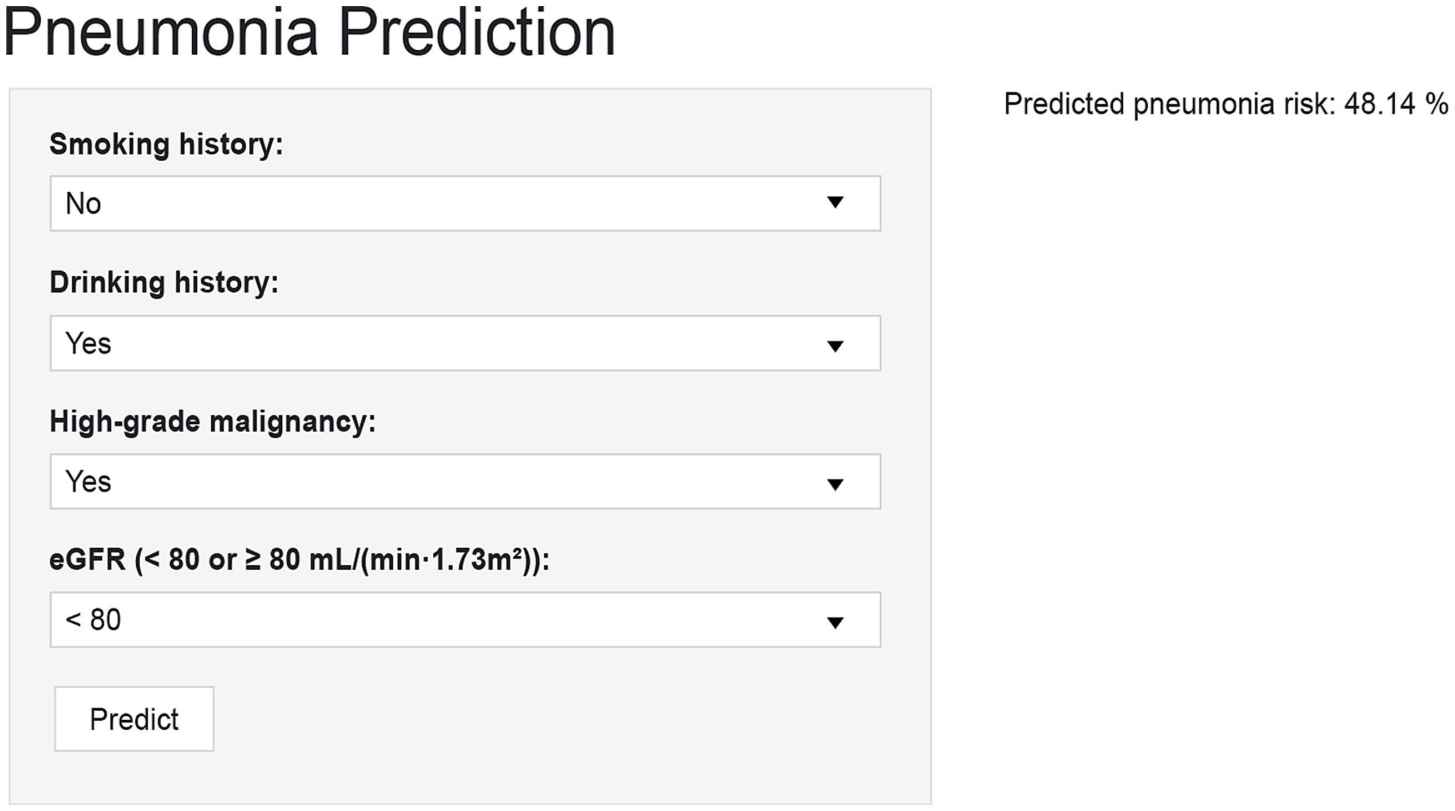
Prototype web-based calculator for individualized pneumonia risk prediction in NHL patients after chemotherapy initiation. The R Shiny interface allows entry of four predictors (smoking, drinking, high-grade malignancy, eGFR) to generate an individualized 90-day pneumonia risk score with interpretive visualization.

To improve clinical applicability, the final GBM model was implemented as an interactive web-based tool using the R Shiny framework. This online platform allows healthcare providers to input patient-specific clinical parameters and obtain real-time estimates of the probability of pneumonia occurring within 90 days of chemotherapy initiation. The web-based tool interface contains four input fields, namely high-grade malignancy, drinking, eGFR, and smoking (Figure 5). After data entry, the system promptly computes and displays the predicted probability of pneumonia, providing a quantitative reference to support clinical evaluation. The tool is accessible through standard web browsers on both desktop and mobile devices, without the need for additional software installation. Its real-time output and integration of SHAP-based interpretive visualizations enhance transparency and support individualized risk assessment in routine hematology practice. The tool is available at: https://ltfu-zzn.shinyapps.io/Pneumonia/

## 4 Discussion

### 4.1 Principal findings

In this study, we developed a machine learning-based model to predict early pneumonia in patients with NHL undergoing chemotherapy. The model was constructed using four clinically relevant predictors: high-grade malignancy, drinking status, eGFR, and smoking status. Among the five algorithms evaluated, the GBM model achieved the highest predictive performance. On the internal hold-out test set, it yielded an AUC of 0.855, an F1 score of 0.679, and a Brier score of 0.155, demonstrating favorable discrimination, calibration, and overall predictive accuracy. Among the evaluated algorithms, GBM achieved a favorable sensitivity–specificity balance and maintained consistent performance across the training set and the internal hold-out test set. The use of SHAP values further facilitated the interpretation of model predictions at both the global and individual levels. Impaired renal function, and behavioral factors such as smoking and drinking were identified as key contributors to pneumonia risk. These findings support the utility of interpretable machine learning techniques in predicting early pneumonia risk in NHL patients, offering a potential tool for personalized risk stratification and preventive care in hematologic oncology. Because our evaluation used an internal hold-out split from the same single-center cohort, true external generalizability remains untested. We therefore plan to conduct temporal validation in a later patient cohort and pursue multicenter external validation to assess transportability; if dataset shift is observed, model recalibration and threshold re-specification will be performed.

### 4.2 Clinical and scientific implications

A previous study reported that 39.7% of newly diagnosed NHL patients developed bacterial pulmonary infections (4). After chemotherapy, the risk of such infections in NHL patients increases significantly. Other studies have shown that most severe infections occur within six months of initiating rituximab-based chemotherapy (33), emphasizing the need for early and continuous monitoring. Such infections frequently lead to treatment delays or discontinuation and were associated with substantially poorer progression-free survival. Accurate assessment of infection risk in the early treatment phase therefore enables timely implementation of preventive measures, including prophylactic antibiotics, enhanced monitoring, and tailored chemotherapy regimens.

Beyond positioning our work relative to MASCC, prior interpretable ML studies in related populations help contextualize our contribution. In lung cancer, Sun et al. predicted post-chemotherapy lung infection using 36 clinical/laboratory predictors selected by Boruta/LASSO; a regularized logistic-regression model with SHAP achieved an AUC ≈ of 0.89 and reported calibration and decision-curve analyses. Their endpoint required clinical signs plus a CT-identified infectious focus, but was not restricted to radiographically confirmed pneumonia within a prespecified 90-day window (34). In newly diagnosed multiple myeloma, Peng et al. trained models on 38 variables and found XGBoost performed best (AUC ≈ 0.88 in validation), again with SHAP explanations; the outcome was a composite infection definition (microbiologically or clinically defined infection, or FUO) rather than pneumonia specifically (35). Both studies emphasized discrimination and interpretability using internal resampling for validation. By contrast, our study focuses on a narrow, clinically actionable endpoint—radiographically confirmed pneumonia within 90 days of chemotherapy initiation in NHL—and demonstrates competitive performance through a parsimonious, interpretable gradient boosting machine model explained by SHAP, incorporating four predictors (high-grade malignancy, eGFR, smoking, and drinking).

This focus is supported by real-world data indicating that nearly half of respiratory complications in NHL occur during this early treatment window, with approximately 75% being infectious in origin (6). The median onset of interstitial pneumonia at 74 days after treatment initiation further illustrates the vulnerability of this phase (36), even though interstitial pneumonia itself is non-infectious. In line with previous studies, smoking was reaffirmed as a risk factor for pneumonia, while high-grade malignancy also emerged as a predictor, plausibly reflecting immune dysfunction in aggressive subtypes such as DLBCL (37). Retrospective analyses of DLBCL have shown high rates of infectious episodes (38) and substantially reduced five-year survival in patients who developed pneumonia during chemotherapy (41% vs. 82%) (39). Similarly, reduced eGFR was associated with pneumonia risk, underscoring the contribution of renal dysfunction during immunochemotherapy (40). Alcohol use was another significant predictor, consistent with meta-analytic evidence linking alcohol consumption to impaired pulmonary immune defenses (41).

Taken together, this study represents a novel application of interpretable ML for pneumonia risk management in NHL. By leveraging GBM with SHAP explanations and decision-curve analysis, we provide not only a high-performing predictive model but also a transparent, clinician-friendly tool. While SHAP offers individualized, transparent rationale for risk estimates, it reflects associations rather than causation; therefore, explanations should be used to inform preventive vigilance and shared decision-making, with thresholds and calibration subject to confirmation in external/temporal validation. Unlike conventional scoring systems, our model captures non-linear relationships and interactions among malignancy severity, disease stage, renal function, and behavioral factors, thereby offering a practical and data-driven approach to pneumonia surveillance in hematologic malignancies.

### 4.3 Comparison with existing risk models

Several clinical scoring tools have been used to assess infection risk in cancer patients, with the MASCC score being one of the most commonly used. However, it was originally developed for febrile neutropenia and may not be well suited to predicting pneumonia, especially in lymphoma patients. The score is based on general clinical parameters, such as the burden of illness and outpatient status, but lacks consideration of tumor biology or treatment-specific factors (42, 43). Our model, in contrast, was developed specifically for NHL patients receiving chemotherapy, with a focus on predicting pneumonia during the early, high-risk phase of treatment. By including variables such as eGFR and high-grade malignancy, it achieved strong performance (AUC = 0.855 on the internal hold-out test set) and may offer advantages over conventional additive scores. Unlike conventional tools that rely on additive point systems and assume linear effects, our GBM model can account for complex interactions among clinical features. Importantly, the use of SHAP values allows the model to explain individual predictions—something traditional scores do not provide (44). Overall, our work offers a more focused and clinically adaptable approach to pneumonia risk assessment in hematologic oncology, complementing existing models while addressing some of their key limitations.

### 4.4 Limitations

This study has several limitations that should be acknowledged. First, this is a single-center study with internal hold-out validation only; performance estimates may therefore be optimistic, and generalizability remains to be confirmed. Given the small internal test set (*n* = 60), the CIs are necessarily wide. Future work will include temporal validation within our center and external, multicenter validation, with attention to site-level distribution shifts and the potential need for recalibration and threshold re-specification; larger validation cohorts should also allow narrower CIs. Second, train-test comparability was assessed using SMDs; several laboratory variables (WBC, ANC, PLT, and CRP) showed modest imbalance (|SMD| ≈ 0.21–0.39). Although none of these variables entered the final four-predictor model (high-grade malignancy, drinking, eGFR, smoking), we also conducted a prespecified sensitivity analysis in which all variables with |SMD| > 0.20 were excluded prior to preprocessing, and we reran the same pipeline. Performance was essentially unchanged relative to the primary analysis (Supplementary Tables S9, S10), suggesting limited impact of this imbalance. Nevertheless, residual bias in internal metrics cannot be fully excluded. Third, phenotype ascertainment may be constrained by the lack of systematic microbiological confirmation and by the absence of centralized, standardized radiologic re-reads/scoring. Although case adjudication was performed by clinicians based on clinical, laboratory, and imaging findings, incorporating detailed information such as pathogen identification or standardized radiographic scoring could enhance diagnostic accuracy and model performance. Fourth, despite performing feature selection and hyperparameter tuning strictly within training cross-validation folds, some risk of overfitting remains. Given the initial 35 candidates and 79 events, selection-induced instability is possible; EPV constraints, the plateau rule, nested-CV stability analyses, and LASSO bootstrapping mitigate—but do not eliminate—this risk. External (temporal/multicenter) validation will be needed to assess feature robustness and the need for recalibration, and threshold re-specification. Finally, the web tool is a prototype for internal validation and has not undergone prospective clinical implementation or impact evaluation.

Taken together, these limitations—particularly the lack of systematic microbiological confirmation, the absence of standardized radiologic re-reads/scoring, and the absence of external (temporal/multicenter) and prospective/real-world validation—substantially limit the immediate clinical translation of our findings. The model should be regarded as research-only pending external validation, potential recalibration and threshold re-specification under dataset shift, and prospective impact evaluation.

### 4.5 Future perspectives

Future work will prioritize temporal and external validation across broader populations and care settings. Multicenter collaborations will be essential to evaluate generalizability and transportability, mitigate overfitting, and monitor calibration drift with protocolized threshold recalibration in new cohorts. Integration with electronic health records—for research use only—will be explored to enable real-time risk scoring, subject to governance, privacy safeguards, and workflow co-design. As healthcare ML matures, future iterations may incorporate longitudinal clinical trajectories, pathogen-specific data, treatment exposures, and imaging-derived features to improve performance while maintaining interpretability.

## 5 Conclusion

We developed and internally validated a gradient-boosting model to estimate the 90-day risk of pneumonia after chemotherapy in NHL using routinely available predictors, with SHAP-based explanations and a research-prototype web tool. Performance on repeated cross-validation and an internal hold-out test set was encouraging; however, the single-center, retrospective design and the modest test sample warrant cautious interpretation. The tool is not intended for clinical decision-making, and external (including temporal and multicenter) validation is required before clinical deployment. With appropriate validation and updates to calibration and threshold specification, this approach may enable earlier identification of higher-risk patients and support targeted preventive strategies. All code is openly available (GitHub: https://github.com/zzn-project/NHL-Pneumonia-ML) to facilitate transparency and reproducibility.

## Data Availability

The raw data supporting the conclusions of this article will be made available by the authors, without undue reservation.

## Glossary

NHL: Non-Hodgkin Lymphoma
RF-RFE: Random Forest-based Recursive Feature Elimination
DLBCL: Diffuse Large B-cell Lymphoma
ML: Machine Learning
LR: Logistic Regression
SVM: Support Vector Machine
KNN: k-Nearest Neighbors
GBM: Gradient Boosting Machine
LightGBM: Light Gradient Boosting Machine
AUC: Area Under the Curve
DCA: Decision Curve Analysis
SHAP: Shapley Additive Explanations
NCCN-IPI: National Comprehensive Cancer Network-International Prognostic Index
WBC: White blood cell count
Hb: Hemoglobin
ANC: Absolute neutrophil count
ALC: Absolute lymphocyte count
RDW: Red cell distribution width
PLT: Platelet count
MPV: Mean platelet volume
PCT: Plateletcrit
eGFR: Estimated glomerular filtration rate
Ca: Calcium
Fe: Iron
ALB: Albumin
A/G ratio: Albumin/globulin ratio
ALT: Alanine aminotransferase
AST: Aspartate aminotransferase
LDH: Lactate dehydrogenase
*α*-HBDH: α-Hydroxybutyrate dehydrogenase
TG: Triglycerides
LDL: Low-density lipoprotein cholesterol
CRP: C-reactive protein
