# Supplementary material for: Interpretable machine learning model to predict 90-day radiographically confirmed pneumonia after chemotherapy initiation in non-Hodgkin lymphoma: development and internal validation of a single-center cohort
Source: Front Med (Lausanne). 2025 Sep 22;12:1674896. doi: 10.3389/fmed.2025.1674896 (PMC12497835; doi:10.3389/fmed.2025.1674896)
Supplement: Supplementary file 1 [file Data_Sheet_1.PDF]

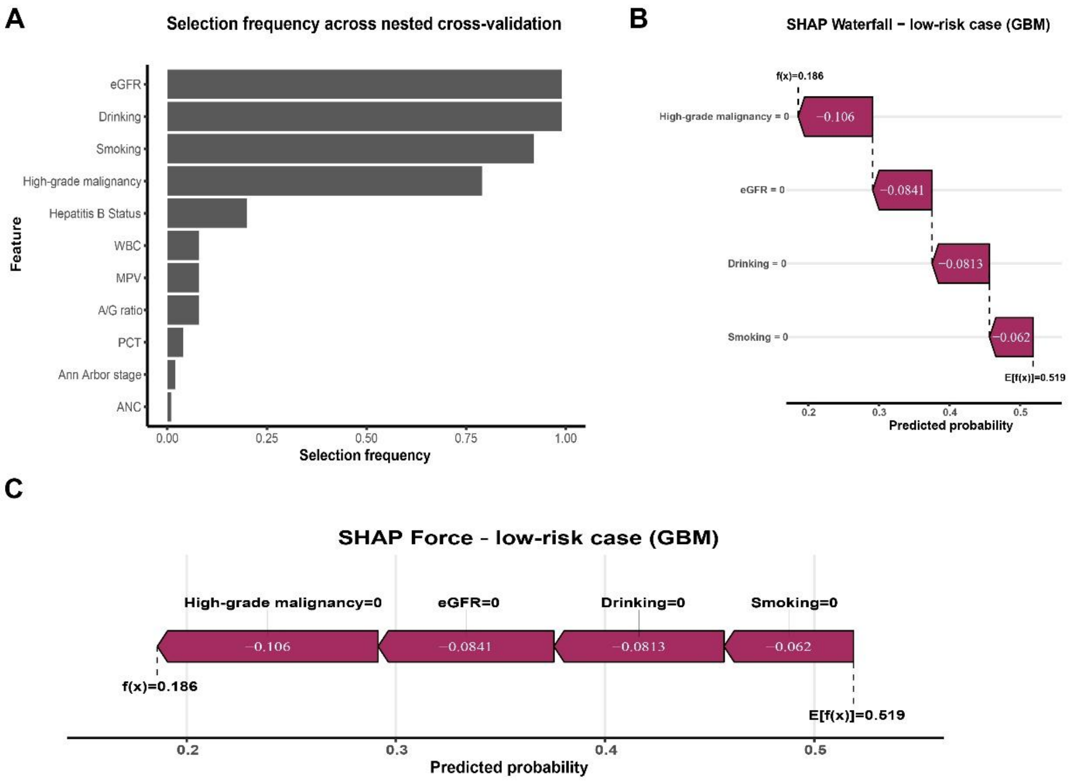

Supplementary Figure S1. Additional feature selection and SHAP-based case-level interpretation.

(A) Selection frequency of candidate predictors across nested cross-validation (outer 5-fold, 20 repeats). eGFR, drinking, smoking, and high-grade malignancy showed the highest stability, consistent with the final feature set.

(B) SHAP waterfall plot for a representative low-risk patient (Smoking = 0, Drinking = 0, eGFR = 0, High-grade malignancy = 0). Negative contributions from four predictors shift the estimated risk downward, resulting in a final predicted probability of 0.186 (baseline E[f(x)] = 0.519).

(C) SHAP force plot for the same low-risk case, illustrating how each predictor contributes to lowering the overall prediction.

Predictor encoding: Smoking (1 = Yes, 0 = No), Drinking (1 = Yes, 0 = No), High-grade malignancy (1 = Yes, 0 = No), eGFR (1 = < 80 mL/min/1.73 m2, 0 = ⩾ 80 mL/min/1.73 m2).

Supplementary Table S1. Summary of variable-wise missingness in the analytic dataset.

Variable	Missing, n	Missing, %
Infection	0	0
Gender	0	0
Age	0	0
Ann Arbor stage	5	2.44
NCCN-IPI	17	8.30
Post-chemotherapy marrow	0	0
Hepatitis B status	0	0
Smoking	0	0
Drinking	0	0
Family history of malignant	0	0
BMI	4	1.95
Hypertension	5	2.44
Diabetes	5	2.44
High-grade malignancy	0	0
Treatment regimen	0	0
WBC	1	0.49
Hb	0	0
ANC	0	0
ALC	0	0
RDW	0	0
PLT	0	0
MPV	0	0
PCT	0	0
eGFR	0	0
Ca	0	0
Fe	0	0
ALB	0	0
A/G ratio	0	0
ALT	0	0
AST	0	0
LDH	4	1.95
α-HBDH	7	3.42
TG	1	0.49
HDL	1	0.49
LDL	1	0.49
CRP	7	3.42

Supplementary Table S2. Stepwise subsets from EPV-constrained RF-RFE after LASSO (λ_{1se}) selection: cross-validated AUCs by subset size. Four predictors were chosen per the pre-specified rule.

Number of	Cross-validated AUC	Feature added at step	Step
2	0.748	Drinking	2
3	0.803	eGFR	3
4	0.804	High-grade malignancy	4

Supplementary Table S3. Selection frequency of predictors across nested cross-validation (outer 5-fold, 20 repeats).

Feature	Selection frequency
Drinking	0.99
eGFR	0.99
Smoking	0.92
High-grade	0.79
Hepatitis B status	0.2
WBC	0.08
MPV	0.08
A/G ratio	0.08
PCT	0.04
Ann Arbor stage	0.02
ANC	0.01

Supplementary Table S4. Bootstrap selection probabilities of candidate predictors based on 200 replications of the LASSO (λ_{1se}) procedure.

Feature	Selection Frequency
eGFR	0.985
Drinking	0.97
Smoking	0.895
High-grade malignancy	0.865
Hepatitis B status	0.705
A/G ratio	0.530
MPV	0.500
PCT	0.500
Ann Arbor stage	0.390
WBC	0.340
ANC	0.310
ALT	0.310
HDL	0.250
ALB	0.205
BMI	0.140
Post-chemotherapy marrow	0.135
Gender	0.125
Hb	0.125
PLT	0.125
TG	0.125
RDW	0.120
ALC	0.110
Diabetes	0.105
Hypertension	0.100
α-HBDH	0.100
LDL	0.100
Age	0.090
NCCN-IPI	0.090
Fe	0.085
Family history of malignant	0.080
AST	0.080
Treatment regimen	0.075
LDH	0.060
CRP	0.055
Ca	0.030

Supplementary Table S5. Variance inflation factors (VIF) for the final predictors in the pneumonia risk model.

Predictor	VIF
eGFR	1.23
Smoking	1.45
Drinking	1.47
High-grade	1.10

Supplementary Table S6. Final hyperparameter settings and training-set decision thresholds (Youden's J) used for model evaluation. Footnote: For each algorithm (logistic regression, SVM, KNN, GBM, LightGBM), the table lists the final parameter values used to train the models in the main text and the training-set decision threshold. All resampling and preprocessing (imputation; SMOTE; centering/scaling for SVM/KNN) were performed within folds on the training data only; the internal hold-out test set was never used for tuning.

Model	Parameter	Value
Logistic	Train-set threshold (Youden's J)	0.386
Logistic	parameter	none
SVM	C	2.264
SVM	Train-set threshold (Youden's J)	0.398
SVM	Sigma	0.001
GBM	Train-set threshold (Youden's J)	0.418
GBM	interaction.depth	4
GBM	n.minobsinnode	19
GBM	n.trees	283
GBM	shrinkage	0.03
KNN	Train-set threshold (Youden's J)	0.167
KNN	distance	1
KNN	kernel	rectangular
KNN	kmax	44
LightGBM	Train-set threshold (Youden's J)	0.386
LightGBM	bagging_fraction	0.737
LightGBM	bagging_freq	1
LightGBM	feature_fraction	0.7
LightGBM	lambda_l1	1.429
LightGBM	lambda_l2	3.090
LightGBM	learning_rate	0.114
LightGBM	min_data_in_leaf	18
LightGBM	nrounds	16
LightGBM	num_leaves	50

Supplementary Table S7. Training-set performance with pre-specified thresholds and results on the internal hold-out test set. Footnote: Thresholds were pre-specified on the training set by maximizing Youden's J (positive class = "Yes") and fixed for testing. AUC 95% CIs by DeLong; other 95% CIs by class-stratified bootstrap (B = 2,000). Counts are TP/TN/FP/FN at the fixed threshold.

Model	Threshold	Accuracy (95% CI)	Sensitivity (95% CI)	Specificity (95% CI)	PPV (95% CI)	NPV (95% CI)	F1 (95% CI)	AUC (95% CI)	TP	TN	FP	FN
Logistic	0.386	0.793 (0.724-0.855)	0.786 (0.679-0.893)	0.798 (0.708-0.876)	0.710 (0.623-0.810)	0.855 (0.795-0.918)	0.679 (0.545-0.792)	0.844 (0.732 – 0.957)	44	71	18	12
SVM	0.398	0.793 (0.724-0.855)	0.786 (0.679-0.893)	0.798 (0.708-0.876)	0.710 (0.623-0.810)	0.855 (0.795-0.918)	0.679 (0.545-0.792)	0.841 (0.729 – 0.953)	44	71	18	12
GBM	0.418	0.793 (0.724-0.855)	0.786 (0.679-0.893)	0.798 (0.708-0.876)	0.710 (0.623-0.810)	0.855 (0.795-0.918)	0.679 (0.545-0.792)	0.855 (0.746 – 0.964)	44	71	18	12
KNN	0.167	0.662 (0.593-0.731)	0.946 (0.875-1.000)	0.483 (0.382-0.584)	0.535 (0.485-0.593)	0.935 (0.860-1.000)	0.629 (0.563-0.697)	0.588 (0.451 – 0.724)	53	43	46	3
LightGBM	0.386	0.793 (0.731-0.855)	0.768 (0.661-0.875)	0.809 (0.719-0.888)	0.717 (0.629-0.818)	0.847 (0.787-0.910)	0.706 (0.571-0.824)	0.841 (0.729 – 0.953)	43	72	17	13

Supplementary Table S8. Test-set performance under an alternative specification varying k in kNN imputation (k = 3, 5, 7). Footnote: Test-set metrics are shown for different values of k used in kNN imputation. Decision thresholds were pre-specified on the training set by maximizing Youden's J (positive class = "Yes," corresponding to pneumonia) and then applied unchanged to the test set. Because this specification differs from the primary modeling pipeline (settings and implementation), the absolute performance values are not intended for direct comparison with the primary test-set results. Results were qualitatively consistent across k values, supporting the robustness of the imputation approach.

k	Training CV	Test AUC	Test	Test	Test	Threshold (Youden from train)
3	0.762	0.689	0.667	0.609	0.703	0.333
5	0.764	0.682	0.667	0.609	0.703	0.330
7	0.762	0.678	0.667	0.609	0.703	0.356

Supplementary Table S9. Sensitivity analysis excluding variables with |SMD| > 0.20 between training and test sets: internal test-set performance (primary pipeline unchanged).

Model	Threshold	Accuracy (95% CI)	Sensitivity (95% CI)	Specificity (95% CI)	PPV (95% CI)	NPV (95% CI)	F1 (95% CI)	AUC (95% CI)	TP	TN	FP	FN
Logistic	0.386	0.717 (0.600--0.817)	0.783 (0.609--0.957)	0.676 (0.514--0.811)	0.600 (0.484--0.731)	0.833 (0.714--0.957)	0.679 (0.545--0.792)	0.844 (0.732--0.957)	18	25	12	5
SVM	0.398	0.717 (0.600--0.817)	0.783 (0.609--0.957)	0.676 (0.514--0.811)	0.600 (0.484--0.731)	0.833 (0.714--0.957)	0.679 (0.545--0.792)	0.841 (0.729--0.953)	18	25	12	5
GBM	0.418	0.717 (0.600--0.817)	0.783 (0.609--0.957)	0.676 (0.514--0.811)	0.600 (0.484--0.731)	0.833 (0.714--0.957)	0.679 (0.545--0.792)	0.855 (0.746--0.964)	18	25	12	5
KNN	0.167	0.567 (0.467--0.667)	0.957 (0.870--1.000)	0.324 (0.189--0.486)	0.468 (0.413--0.537)	0.923 (0.750--1.000)	0.629 (0.563--0.697)	0.588 (0.451--0.724)	22	12	25	1
LightGBM	0.386	0.750 (0.633--0.850)	0.783 (0.609--0.957)	0.730 (0.568--0.865)	0.643 (0.516--0.783)	0.844 (0.735--0.962)	0.706 (0.571--0.824)	0.841 (0.729--0.953)	18	27	10	5

Supplementary Table S10.Sensitivity analysis excluding variables with |SMD| > 0.20 between training and test sets: training-set performance (primary pipeline unchanged).

Model	Threshold	Accuracy (95% CI)	Sensitivity (95% CI)	Specificity (95% CI)	PPV (95% CI)	NPV (95% CI)	F1 (95% CI)	AUC (95% CI)	TP	TN	FP	FN
Logistic	0.386	0.793 (0.724-0.855)	0.786 (0.679-0.893)	0.798 (0.708-0.876)	0.710 (0.623-0.810)	0.855 (0.795-0.918)	0.679 (0.545-0.792)	0.844 (0.732 – 0.957)	44	71	18	12
SVM	0.398	0.793 (0.724-0.855)	0.786 (0.679-0.893)	0.798 (0.708-0.876)	0.710 (0.623-0.810)	0.855 (0.795-0.918)	0.679 (0.545-0.792)	0.841 (0.729 – 0.953)	44	71	18	12
GBM	0.418	0.793 (0.724-0.855)	0.786 (0.679-0.893)	0.798 (0.708-0.876)	0.710 (0.623-0.810)	0.855 (0.795-0.918)	0.679 (0.545-0.792)	0.855 (0.746 – 0.964)	44	71	18	12
KNN	0.167	0.662 (0.593-0.731)	0.946 (0.875-1.000)	0.483 (0.382-0.584)	0.535 (0.485-0.593)	0.935 (0.860-1.000)	0.629 (0.563-0.697)	0.588 (0.451 – 0.724)	53	43	46	3
LightGBM	0.386	0.793 (0.731-0.855)	0.768 (0.661-0.875)	0.809 (0.719-0.888)	0.717 (0.629-0.818)	0.847 (0.787-0.910)	0.706 (0.571-0.824)	0.841 (0.729 – 0.953)	43	72	17	13
